# Effects of vitamin D administration on nociception and spinal cord pro-oxidant and antioxidant markers in a rat model of neuropathic pain

**DOI:** 10.1590/1414-431X2021e11207

**Published:** 2021-08-06

**Authors:** M.C.Q. Santos, T.C.B. da Silva, F.B.O. da Silva, C. Siebert, A. Kroth, E.M.S. Silveira, A.T.S. Wyse, W.A. Partata

**Affiliations:** 1 Laboratório de Neurobiologia Comparada, Departamento de Fisiologia, Instituto de Ciências Básicas da Saúde, Universidade Federal do Rio Grande do Sul, Porto Alegre, RS, Brasil; 2 Departamento de Bioquimica, Instituto de Ciências Básicas da Saúde, Universidade Federal do Rio Grande do Sul, Porto Alegre, RS, Brasil; 3 Área Ciências da Vida, Universidade do Oeste de Santa Catarina, Joaçaba, SC, Brasil

**Keywords:** Total thiol content, Superoxide anion generation, Total antioxidant capacity, Sciatic functional index, Mechanical withdrawal threshold, Thermal withdrawal latency

## Abstract

Reactive oxygen species (ROS) are involved in neuropathic pain, a complicated condition after nerve tissue lesion. Vitamin D appears to improve symptoms of pain and exhibits antioxidant properties. We investigated the effects of oral administration of vitamin D_3_, the active form of vitamin D, on nociception, the sciatic functional index (SFI), and spinal cord pro-oxidant and antioxidant markers in rats with chronic constriction injury (CCI) of the sciatic nerve, a model of neuropathic pain. Vitamin D_3_ (500 IU/kg per day) attenuated the CCI-induced decrease in mechanical withdrawal threshold and thermal withdrawal latency (indicators of antinociception) and SFI. The vitamin prevented increased lipid hydroperoxide levels in injured sciatic nerve without change to total antioxidant capacity (TAC). Vitamin D_3_ prevented increased lipid hydroperoxide, superoxide anion generation (SAG), and hydrogen peroxide (H_2_O_2_) levels in the spinal cord, which were found in rats without treatment at 7 and 28 days post-CCI. A significant negative correlation was found between mechanical threshold and SAG and between mechanical threshold and H_2_O_2_ at day 7. Vitamin D_3_ also prevented decreased spinal cord total thiols content. There was an increase in TAC in the spinal cord of vitamin-treated CCI rats, compared to CCI rats without treatment only at 28 days. No significant changes were found in body weight and blood parameters of hepatic and renal function. These findings demonstrated, for first time, that vitamin D modulated pro-oxidant and antioxidant markers in the spinal cord. Since antinociception occurred in parallel with oxidative changes in the spinal cord, the oxidative changes may have contributed to vitamin D-induced antinociception.

## Introduction

Neuropathic pain is caused by a lesion or disease of the somatosensory nervous system, which occurs in a variety of pathological conditions including trauma, diabetes, cancer, multiple sclerosis, ischemic attack, alcoholism, spinal cord damage, and many others (1). The prevalence of this type of pain is estimated at 1.5 to 8% of the general population, but the incidence is likely to increase due to the ageing global population, higher incidence of diabetes mellitus, and increased survival from cancer after chemotherapy (1).

Different mechanisms are involved in neuropathic pain, including reactive oxygen species (ROS) (2). ROS comprise both free radical and non-free radical oxygen-containing molecules such as superoxide radical and hydrogen peroxide (H_2_O_2_) (3). Studies have demonstrated that superoxide appears to mediate long-term potentiation in excitatory neurons and long-term depression in inhibitory interneurons of the spinal cord in neuropathic pain (4). H_2_O_2_ was found to activate the TRPM2 channel, a calcium permeable cation channel, in dorsal root ganglia neurons of rats with neuropathic pain induced by diabetes (5).

Neuropathic pain also changes antioxidant systems (2), which minimize the levels of ROS while allowing for their useful roles in cell signaling and redox regulation (3). Thiol compounds are a natural reservoir of reductive capacity of cells (6) and studies have demonstrated that glutathione, the most abundant thiol in mammals, is reduced in nerve and spinal cord of rats with neuropathic pain (7,8).

The complex pathogenesis of neuropathic pain contributes to its poor treatment. Studies have highlighted the limited efficacy of clinical therapeutic drugs and the presence of long-term side effects of drugs used for neuropathic pain management (1). This difficulty has led to the search for new therapeutic strategies in order to find effective drugs to treat neuropathic pain with minimal, or at least reduced, adverse effects.

Vitamin D appears to play an important role in pain. Supplementation with this vitamin reduced pain scores in patients with different painful conditions (9). There is an increasing number of studies indicating that vitamin D deficiency appears to be associated with various forms of chronic pain (9,10). Administration of vitamin D_3_, the biologically active form of vitamin D, attenuated behavioral scores of neuropathic pain in rats with chronic constriction injury (CCI) of the sciatic nerve (11), a model that simulates the symptoms of chronic nerve compression, which correspond to causalgia or complex regional pain syndrome in human patients (12).

Vitamin D also appears to play a major role in regulating ROS levels. Studies have shown that this vitamin takes part in regulating the expression of many antioxidant systems that prevent oxidative stress by removing ROS and also by reversing the oxidative changes that occur during normal ROS signaling (13–15). A recent systematic review and meta-analysis of clinical trials showed that vitamin D supplementation increases total antioxidant capacity and glutathione, and decreases lipid peroxidation in blood, with the latter being a marker of damage to lipids caused by ROS (16).

Therefore, we hypothesized that the antinociceptive effect of vitamin D would involve changes in pro-oxidant and antioxidant markers in the sciatic nerve and in the region of the spinal cord where the afferent fibers of the sciatic nerve enter. Thus, the present study assessed, for the first time, the relationship between vitamin D_3_-induced antinociception and alterations in sciatic nerve and spinal cord pro-oxidant and antioxidant markers in rats with CCI-induced neuropathic pain. These findings will have important implications not only by enhancing the understanding of the mechanisms involved in the analgesic effect of vitamin D, but also by pointing towards the potential use of this vitamin in the management of neuropathic pain.

## Material and Methods

### Experimental animals and treatment

All experimental procedures were approved by the Ethics Committee for Animal Experimentation of the Universidade Federal do Rio Grande do Sul (CEUA-UFRGS #32295). A total of 72 adult male Wistar rats (8 weeks old, weighing 180-250 g) were used in all the experiments. The rats were randomly and blindly divided into three experimental groups: naive (rats did not undergo surgical manipulation), sham (rats in which all surgical procedures involved in CCI were carried out except ligature), and CCI (rats in which four ligatures were tied loosely around the right common sciatic nerve). Each group was further divided into two treatments, receiving vitamin D_3_ (Sigma Chemical Co., USA) or vehicle (commercially available olive oil, Monde, Argentina) for 7 or 28 days (n=6 rats/treatment). All rats were housed in a room with a stable temperature of 23±1°C and were fed a regular pellet diet *ad libitum*.

**Figure 1:**
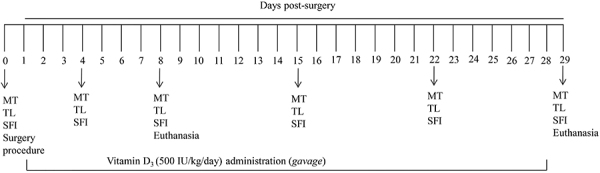
Timeline with days of treatments, behavior tests, and euthanasia of rats. MT: mechanical withdrawal threshold; TL: thermal withdrawal latency; SFI: sciatic functional index.

Vitamin D_3_ was freshly prepared in 0.3 mL olive oil and administrated by gavage at a dose of 500 IU/kg per day. The administration started 24 h after surgery and was performed daily at 5:00 p.m. by the same researcher (11,17,18). Rats were not anesthetized for gavage. The timeline of the experiment is provided in Figure 1.

The dose of vitamin D_3_ was chosen because it showed antinociceptive effects in rats with peripheral nerve injury (11,19). The time periods of the experiment (7 or 28 days) were based on a previous study with vitamin D_3_ administration in CCI rats (11).

### Induction of CCI

Neuropathic pain was induced by CCI according to the model described by Bennett and Xie (20) with slight modifications (7,17). Rats were anesthetized with 90 mg/kg ketamine and 10 mg/kg xylazine, intraperitoneally. The right sciatic nerve was then exposed at the level of the mid-thigh, around which four ligatures (4.0 chromic catgut, Shalon Fios Cirúrgicos Ltda, Brazil) were loosely tied with a 1.0-1.5 mm interval between each. Muscle and skin were sutured and a topical antibiotic (Ryfamicin SV-10 mg/mL, Sanofi-Aventis Farmacêutica Ltda, Brazil) was applied to the wound. After regaining consciousness, the rats were returned to their cages and maintained in the same conditions as previously described. For sham rats, the sciatic nerve was exposed but without ligation, while naive rats did not undergo any surgical manipulation.

### Functional testing

Three standard behavioral tests were employed to assess nociception and sensorimotor deficit in CCI rats: the mechanical analgesiometry, hot plate, and footprint tests. The mechanical analgesiometry and hot plate tests were used to determine mechanical withdrawal threshold and latency to paw withdrawal to thermal stimulus, respectively, as measurements of nociception (7,11,17). The footprint test was used to determine the sciatic functional index (SFI), as evidence of functional recovery post-nerve lesion (18). Rats were habituated to the testing apparatus one day prior to starting experimental tests.

#### Mechanical withdrawal threshold and thermal withdrawal latency

The tests were assessed as described in our previous studies (7,17,18). Mechanical withdrawal threshold was assessed using a digital analgesiometer (Insight, Brazil). A positive response was indicated by an abrupt withdrawal of the injured paw to increasing pressure against the plantar surface. The intensity of the pressure was recorded by the apparatus in grams. The mean of five positive responses in a particular timing trial was considered the threshold for the trial.

Thermal withdrawal latency was assessed by placing the rats on a hot plate maintained at 50°C (±2°C) and measuring paw withdrawal latency. Latency was considered when the animal jumped or licked the hind paw, independently of side. A cutoff time of 30s was employed to prevent tissue injury.

#### SFI

This index was assessed as described by de Medinaceli et al. (21) with some modifications (18). Testing used an apparatus composed of an open field illuminated by a light in which a runway was arranged leading into a dark wooden box. Briefly, the hindpaws of a rat were dyed by pressing them on a black inkpad. The rat was then allowed to walk across a sheet of paper in the illuminated open field leading to the dark box. Rat footprints were used to determine the following measurements, which were obtained from the left (L) and right (R) paws: 1) distance from heel to the third toe (print length, PL); 2) distance from the first to the fifth toe (toe spread, TS); and 3) distance from the second to the fourth toe (intermediate toe spread, ITS). The SFI was calculated as follows: SFI = -38.3 (EPL-RPL) / RPL + 109.5 (ETS-RTS) / RTS + 13.3 (EIT-RIT) / RIT - 8.8

An SFI of zero (±11) indicates normal function, while -100 represents total loss of function. Intermediate results indicate intermediate loss of function. Measurements were made manually and always by two researchers, one of which was blinded to experimental groups.

Rats were subjected to behavioral tests before the surgical procedure and at days 4, 8, 15, 22, and 29 post-surgery, i.e., after 3, 7, 14, 21, and 28 gavages. Tests were conducted at the same time of day (7 a.m.) and by the same researchers, starting with the von Frey test followed by the hot plate test and then the footprint test. Despite no evidence that the tests affected each other, the interval between consecutive tests was ∼30 min for each rat because ≤20 s or ≥20 min were the optimal intervals for consecutive tests in the hot water tail-flick test (22). Two researchers were always present throughout each session, and behavioral test protocols were highly standardized and maintained over time.

### Sample preparation

Body weight was evaluated in all rats that received vehicle and vitamin D_3_ until 28 days and was recorded before the surgical procedure and at days 8, 15, 22, and 29 after CCI, i.e., after day 7, 14, 21, and 28 gavages. All rats were subsequently euthanized by decapitation, and blood, whole lumbosacral spinal cord, and a segment (±7 mm) of the sciatic nerve located above the injury site were promptly collected. Blood was centrifuged at 1000 *g* for 20 min at 4°C and the plasma was used to determine levels of aspartate aminotransferase (AST), alanine aminotransferase (ALT), direct bilirubin, and creatinine using commercially available kits (LABTEST, Brazil). These assessments were made only in naive rats to reveal possible side effects of the treatment.

The spinal cord was divided transversely into three parts. The same portion always received the same preparation. Two parts of the spinal cord were cooled in liquid nitrogen and processed to determine superoxide anion generation (SAG) and H_2_O_2_ levels. The third part was homogenized in 1.15% KCl diluted 1:5 (w/v) containing 1 mM phenylmethylsulfonyl fluoride and centrifuged at 1000 *g* for 20 min at 4°C. The supernatant was then used for assays of lipid hydroperoxide levels, TAC, and total thiol content as done elsewhere (7). The sciatic nerve segment received similar homogenization and the supernatant was used for assays of lipid hydroperoxide levels and TAC.

### Antioxidant parameters

TAC was determined using 2,2-azinobis-(3-ethylbenzothiazoline-6-sulfonic acid) radical cation (ABTS^•+^), which, in an acid medium, is decolorized by antioxidants according to their concentration and antioxidant capacity, as described by Erel (23) and previously used by Riffel et al. (7). This assay was performed with samples of sciatic nerve and spinal cord. Absorbance was read at 660 nm. The assay was carried out in duplicate and replicated twice for each sample. Results are reported in µmol eq trolox/g tissue.

The assay of total thiol content used 30 μL of spinal cord sample, which was mixed with 1 mL of phosphate/EDTA buffer, pH 7.5, and 5,5′-ditiobis (2-nitrobenzoic) acid (DTNB, 10 mM), as described by Aksenov et al. (24) and previously used by Riffel et al. (7). Absorbance was read at 412 nm after 30 min of incubation at room temperature. The assay was carried out in duplicate and replicated at least twice for each sample. Results are reported as mmol/g tissue.

### Pro-oxidant parameters

Lipid hydroperoxides were measured by oxidation of Fe^2+^ in an acid medium containing xylenol orange dye, which forms a complex with Fe^3+^, as described by Jiang et al. (25) and previously used by Riffel et al. (7). This assay was carried out using sciatic nerve and spinal cord samples. Absorbance was read at 560 nm. The assay was replicated at least twice. Results are reported as µmol/g tissue.

Levels of SAG were estimated by the reduced nitroblue tetrazolium (NBT) method described by Wang et al. (26) and previously used by Horst et al. (18). Briefly, sections of fresh lumbosacral spinal cord were reacted with NBT to form formazan as an index of superoxide anion generation. Formazan absorbance was determined spectrophotometrically at 540 nm. The quantity of NBT reduction was calculated as follows: quantity of NBT reduction=A×V/(T×Wt×ε×l), where A is the absorbance of blue formazan at 540 nm, V is the volume of the solution, T is the time period (90 min) during which the samples were incubated with NBT, Wt is the blotted wet weight of the spinal cord portion, ε is the extinction coefficient of blue formazan (i.e., 0.72 L^.^mmol^-1.^mm^-1^), and l is the length of the light path. Results are reported as reduced NBT pmol^.^min^-1.^mg tissue^-1^.

Levels of H_2_O_2_ were measured using horseradish peroxidase (HRPO)-mediated oxidation of phenol red by H_2_O_2_, leading to the formation of a compound that absorbs at 610 nm according to Pick et al. (27) and previously used by Riffel et al. (7). The results are reported as μmol H_2_O_2_/g tissue.

### Statistical analysis

Data were analyzed by two independent researchers, one of which was blind to treatment. All data are reported as means±SE for six animals. Normal Gaussian distribution of the data was analyzed using the Shapiro-Wilk test, while Levene's test was used to analyze homogeneity of variances. Behavioral parameters were compared by repeated-measures ANOVA. Body weight and biochemical results were analyzed using two-way ANOVA (factors: surgery and treatment). Tukey's test was used as the *post hoc* test. Pearson correlation coefficients showed correlations between the von Frey test and SAG and between the von Frey test and H_2_O_2_ at day 8. Differences were considered statistically significant with P<0.05. Statistical analyses were carried out with SigmaPlot software version 11.0 (Systat Software Inc., USA).

## Results

### Functional testing

**Figure 2:**
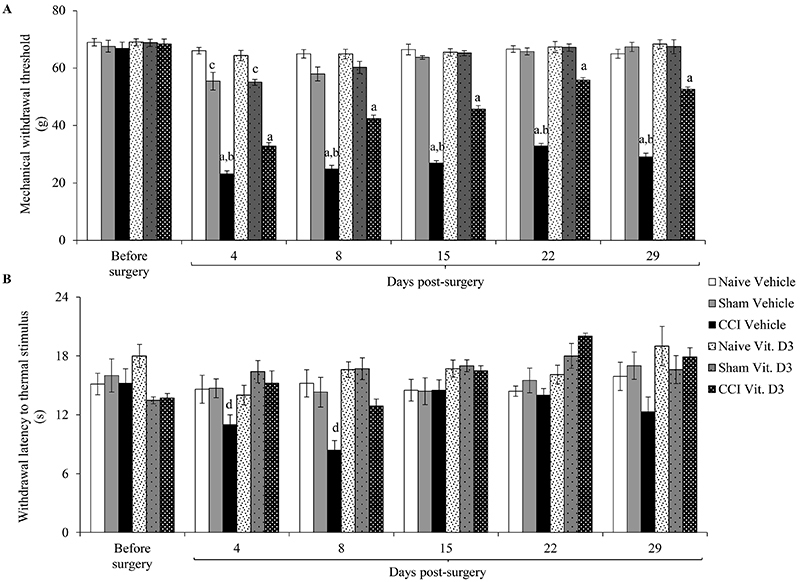
Assessment of mechanical withdrawal threshold (**A**) and withdrawal latency to thermal stimulus (**B**) in rats with chronic constriction injury (CCI) of the sciatic nerve that received vitamin D_3_ (Vit. D3) daily per gavage (500 IU/kg per day) or vehicle for 28 days after surgery. The tests were carried out before surgery and at 4, 8, 15, 22, and 29 days post-surgery. Data are reported as means±SE (n=6/group). ^a^P<0.05 compared to before surgery and naive and sham groups; ^b^P<0.05 compared to vitamin-treated CCI rats at the same measurement time; ^c^P<0.05 compared to before surgery and naive groups; ^d^P<0.05 compared to before surgery (repeated-measures ANOVA followed by Tukey's *post hoc* test). For Sham rats, all surgical procedures involved in the CCI were used except the ligature.

Repeated-measures ANOVA indicated an effect of treatment on mechanical withdrawal threshold (F_2,53_=234.136, P<0.001). Tukey's *post hoc* test revealed that while mechanical withdrawal threshold decreased approximately 67% in vehicle-treated CCI rats, the reduction was approximately 52% in vitamin-treated CCI rats at days 4 and 8 compared to pre-nerve lesion levels and naive and sham rats (Figure 2A). At days 15 and 29, the mechanical withdrawal threshold of vehicle-treated CCI rats decreased by approximately 64 and 56%, respectively, compared to pre-nerve-lesion levels and naive and sham rats. However, the mechanical withdrawal threshold of vitamin-treated CCI rats decreased by 38 and 23% at days 15 and 29, respectively, compared to pre-nerve-lesion levels and naive and sham rats. Thus, vitamin D_3_-treated CCI rats showed an improvement in mechanical withdrawal threshold over vehicle-treated rats of 43% at day 4, approximately 72% at days 8 and 15, and 74% at days 22 and 29.

Sham rats also exhibited decreases in mechanical withdrawal threshold at days 4 and 8 compared to pre-nerve-lesion levels and naive rats. Decreases also occurred in both vitamin D_3_- and vehicle-treated CCI rats, being 20% at day 4, approximately 14.5% at day 8, and no significant changes for the other days. Mechanical threshold did not change significantly in the naive group.

Repeated-measures ANOVA indicated an effect of treatment on thermal withdrawal latency (F_2,53_=2.568, P<0.001). Tukey's *post hoc* test revealed that thermal withdrawal latency significantly decreased in vehicle-treated CCI rats at days 4 and 8 with reductions of 23 and 40%, respectively, compared to pre-nerve-lesion levels. These differences were not found for vitamin D_3_-treated CCI rats (Figure 2B). Vitamin D_3_-treated CCI rats showed an improvement in thermal withdrawal latency over vehicle-treated rats with 39 and 80% at days 4 and 8, respectively. No significant change was found in thermal withdrawal latency for naive and sham rats.

**Figure 3:**
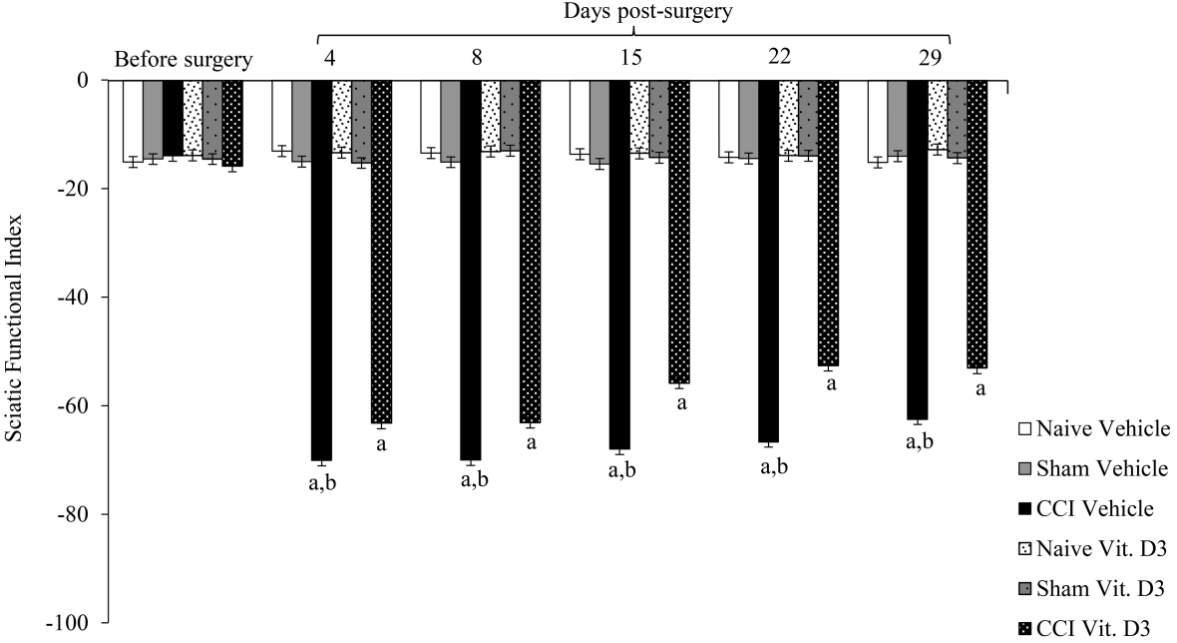
Functional recovery assessed by the sciatic functional index (SFI) in rats with chronic constriction injury (CCI) of the sciatic nerve that received vitamin D_3_ (Vit. D3) daily per gavage (500 IU/kg per day) or vehicle for 28 days after surgery. The tests were carried out before surgery and at 4, 8, 15, 22, and 29 days post-surgery. Data are reported as means±SE (n=6/group). ^a^P<0.05 compared to before surgery and naive and sham groups; ^b^P<0.05 compared to vitamin-treated CCI rats at same measurement time (repeated-measures ANOVA followed by Tukey's *post hoc* test). For Sham rats, all surgical procedures involved in the CCI were used except the ligature.

Repeated-measures ANOVA indicated an effect of treatment on SFI (F_2,53_=3916.512, P<0.001). This index was near zero for naive and sham rats at all measurement times, indicating normal sciatic nerve function. However, all CCI rats experienced a decrease in SFI after surgery (Figure 3). The index was near -100 in all CCI rats at postoperative days 4, 8, 15, 22, and 29, indicating loss of sciatic nerve function. However, SFI differed significantly between vitamin D_3_ and vehicle-CCI rats, with vitamin D_3_ inducing a significant recovery in this index. While SFI recovered only 11% in vehicle-treated CCI rats at 29 days compared to 4 days, vitamin D_3_-treated CCI rats exhibited recoveries of around 10, 17, 21, and 15% at days 4, 15, 22, and 29, respectively, although the vitamin treatment at 28 days did not return SFI to near zero.

### Antioxidant parameters

**Figure 4:**
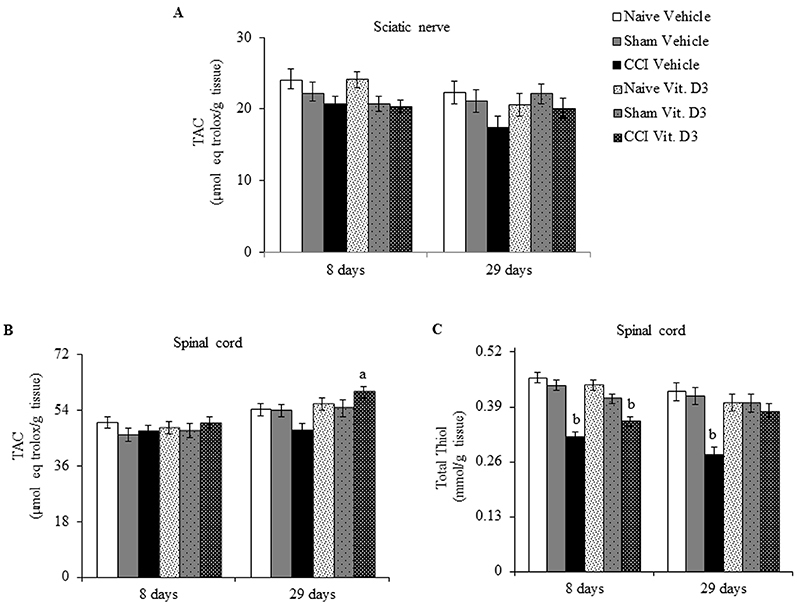
Total antioxidant capacity (TAC) (**A** and **B**) and total thiol content (**C**) in injured sciatic nerve (**A**) and spinal cord (**B** and **C**) of rats treated with vitamin D_3_ (Vit. D3) (500 IU/kg per day) or vehicle for 7 or 28 days after chronic constriction injury (CCI). Rats were euthanized at 8 and 29 days post-surgery. Data are reported as means±SE (n=6/group). ^a^P<0.05 compared to vehicle-treated CCI rats at the same measurement time; ^b^P<0.05 compared to naive and sham groups (two-way ANOVA followed by Tukey's *post hoc* test). For Sham rats, all surgical procedures involved in the CCI were used except the ligature.

Sciatic nerve TAC did not differ significantly among groups (F_2,53_=2.341, P=0.122) or between treatments (F_2,53_=0.281, P=0.602), and with no significant interaction (F_2,53_=1.080, P=0.358) (Figure 4A). A different result was found in spinal cord, with TAC not differing significantly among groups (F_2,53_=0.402, P=0.673) but differing significantly between treatments (F_2,53_=8.737, P=0.007). There was also a significant interaction between group and treatment (F_2,53_=4.689, P=0.019). Tukey's *post hoc* test revealed a significant increase in spinal cord TAC of vitamin-treated CCI rats compared to vehicle-treated CCI rats at day 29 (Figure 4B). However, there was no significant difference in spinal cord TAC between vitamin-treated CCI rats and naive and sham rats. No significant change was found for spinal cord TAC of sham and naive rats.

Spinal cord total thiol content differed significantly among groups (F_2,53_=12.604, P<0.001) but not between treatments (F_2,53_=1.095, P=0.307), with a significant interaction (F_2,53_=7.891, P=0.003). Tukey's *post hoc* test revealed a significant decrease in spinal cord total thiol content of vehicle-treated CCI rats (Figure 4C), with decreases of approximately 29 and 32% at days 8 and 29, respectively, compared to naive and sham rats. However, total thiol content decreased about 20% for the spinal cord of vitamin D_3_-treated CCI rats at day 8, while at day 29 the decrease was only 7% (not significant), compared to naive and sham rats. Thus, vitamin D_3_ administration prevented the decrease in spinal cord total thiol content of CCI rats by around 12 and 36% at days 8 and 29, respectively, compared to vehicle-treated CCI rats. No significant change in spinal cord total thiol content was found for naive and sham rats.

### Pro-oxidant parameters

**Figure 5:**
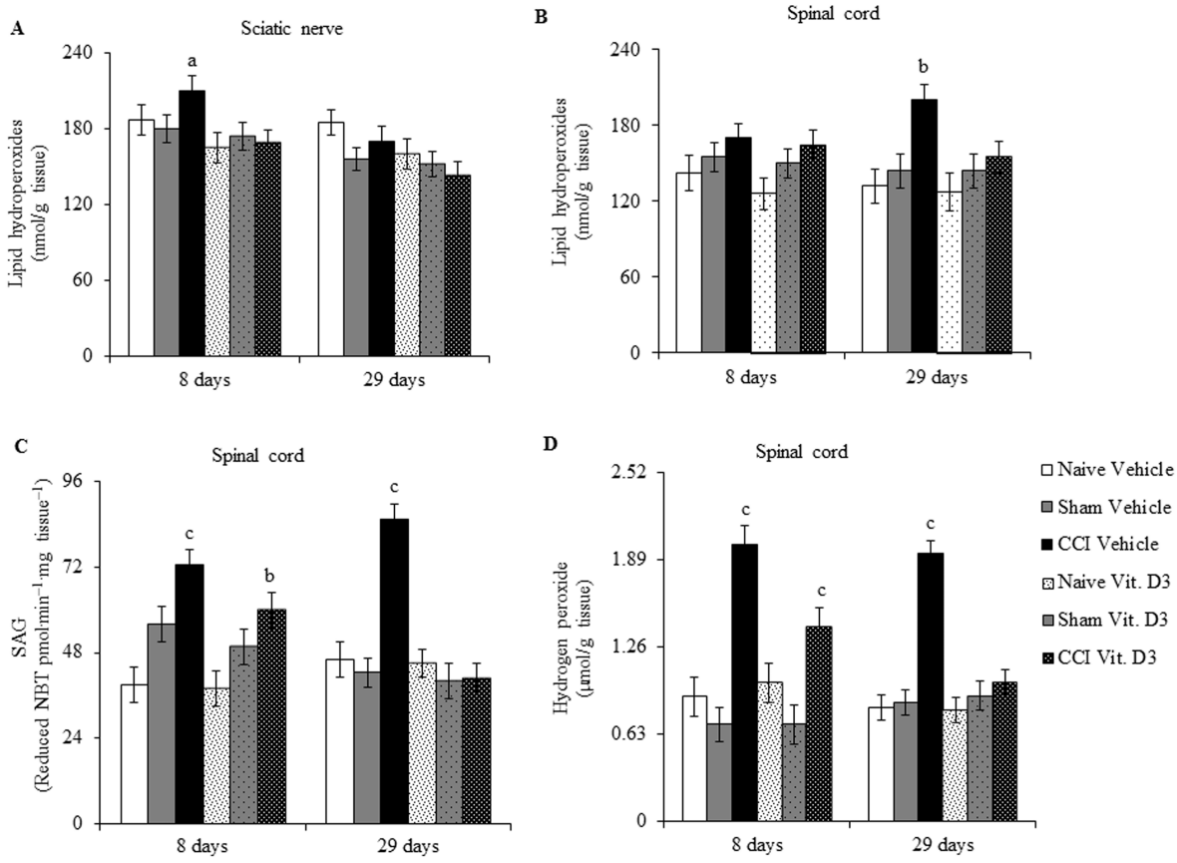
Lipid hydroperoxides (**A** and **B**), superoxide anion generation (SAG) (**C**), and hydrogen peroxide (**D**) levels in injured sciatic nerve (**A**) and spinal cord (**B**-**D**) of rats treated with vitamin D_3_ (Vit. D3) (500 IU/kg per day) or vehicle for 7 or 28 days after chronic constriction injury (CCI). Rats were euthanized at 8 and 29 days post-surgery. Data are reported as means±SE (n=6/group). ^a^P<0.05 compared to vitamin D-treated CCI rats at the same measurement time; ^b^P<0.05 compared to naive groups; ^c^P<0.05 compared to naive and sham groups (two-way ANOVA followed by Tukey's *post hoc* test). For Sham rats, all surgical procedures involved in the CCI were used except the ligature.

Sciatic nerve lipid hydroperoxide levels did not differ significantly among groups (F_2,53_=2.806, P=0.087) but did differ significantly between treatments (F_2,53_=6.794, P=0.018), with no significant interaction (F_2,53_=2.287, P=0.754). Tukey's *post hoc* test showed that lipid hydroperoxide levels increased significantly only in injured sciatic nerve of vehicle-treated CCI rats at day 8 compared to vitamin-treated CCI rats at the same time of measurement (Figure 5A). There was no significant change in lipid hydroperoxide levels in injured sciatic nerve of vitamin D_3_-treated CCI rats and naive and sham rats.

Spinal cord lipid hydroperoxide levels differed significantly among groups (F_2,53_=6.923, P=0.004) but not between treatments (F_2,53_=2.237, P=0.147), with no significant interaction (F_2,53_=1.835, P=0.180). Tukey's *post hoc* test revealed a significant increase in lipid hydroperoxide levels only in spinal cord of vehicle-treated CCI rats at day 29 compared to naive rats (Figure 5B). No significant change was found in lipid hydroperoxide levels in spinal cord of vitamin D_3_-treated CCI rats and sham and naive rats.

Spinal cord SAG differed significantly among groups (F_2,53_=24.241, P<0.001) and between treatments (F_2,53_=31.737, P<0.001), with a significant interaction (F_2,53_=19.756, P<0.001). The level of SAG increased significantly in spinal cord of vehicle-treated CCI rats at days 8 and 29 post-CCI, compared to naive and sham rats. SAG also increased significantly in spinal cord of vitamin-treated CCI rats, but only at day 8 compared to naive rats (Figure 5C). No significant change was found for SAG in spinal cord of vitamin-treated CCI rats at day 29 and no significant change at all was found for SAG in spinal cord of naive and sham rats.

There was a significant negative correlation between SAG in spinal cord and the von Frey test at day 8 (vehicle-treated CCI rats: r=-0.832, P=0.0008; vitamin-treated CCI rats: r=-0.659, P=0.0382).

Spinal cord H_2_O_2_ levels differed significantly among groups (F_2,53_=31.622, P<0.001) and between treatments (F_2,53_=16.948, P<0.001), with a significant interaction (F_2,53_=16.761, P<0.001). Tukey's *post hoc* test revealed that H_2_O_2_ levels increased significantly in spinal cord of vehicle-treated CCI rats at days 8 and 29 compared to naive and sham rats. For vitamin D_3_-treated CCI rats, H_2_O_2_ levels increased significantly only at day 8 compared to naive and sham rats (Figure 5D). No significant change was found for H_2_O_2_ levels in spinal cord of vitamin-treated CCI rats at day 29 and no significant change was found for H_2_O_2_ levels in spinal cord of naive and sham rats.

There was a significant negative correlation between H_2_O_2_ levels in spinal cord and the von Frey test at day 8 (vehicle-treated CCI rats: r=-0.928, P=0.000109; vitamin-treated CCI rats: r=-0.632, P=0.05).

### Effects on blood and hepatic parameters and body weight

**Figure 6:**
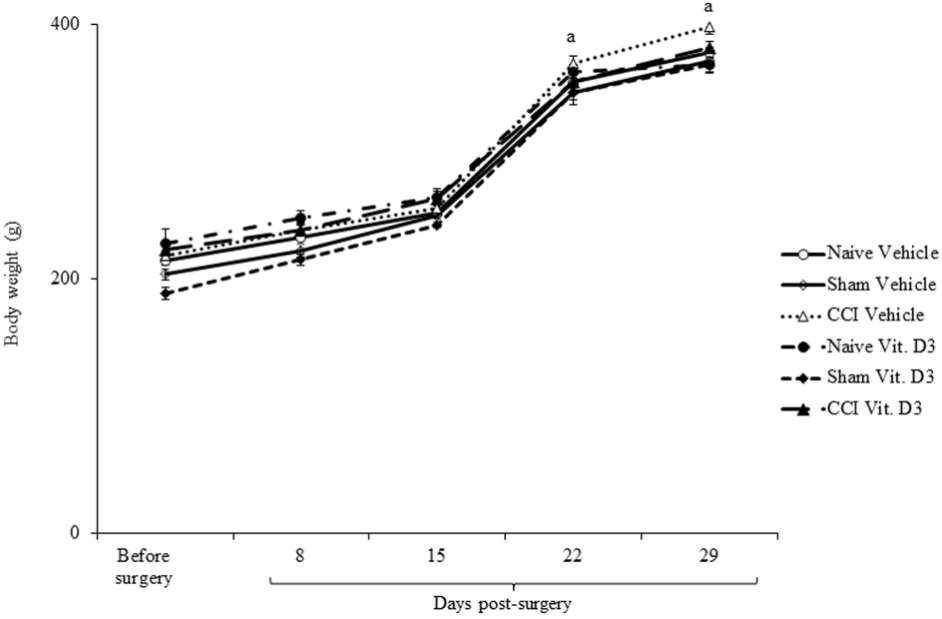
Body weight of rats treated with vitamin D_3_ (Vit. D3) (500 IU/kg per day) or vehicle for 28 days after chronic constriction injury (CCI). Data are reported as means±SE (n=6/group). ^a^P<0.05 compared to values obtained before lesion and days 8 and 15 after surgery (two-way ANOVA followed by Tukey's *post hoc* test). For Sham rats, all surgical procedures involved in the CCI were used except the ligature.

Plasmatic AST, ALT, direct bilirubin, and creatinine levels showed no significant changes with administration of vitamin D_3_ (Table 1). ANOVA showed significant differences in body weight at days 22 and 29 compared to before surgery and days 8 and 15 after surgery (F_2,53_=140.058, P<0.001). However, there was no significant difference in body weight between treatments. Rats that received vitamin D_3_ for 28 days (day 29 after CCI) continued to grow and gain weight similarly to vehicle-treated rats (Figure 6).

**Table 1:** Effect of vitamin D_3_ (Vit. D_3_) administration on plasmatic parameters in naive rats.

	Vehicle	Vit. D_3_
AST (U/L)	175.4±9.86	163.5±13.40
ALT (U/L)	43.7±7.00	36.9±11.20
Direct bilirubin (mg/dL)	0.81±0.08	0.74±0.06
Creatinine (mg/dL)	0.74±0.10	0.61±0.06

Data are reported as means±SE (n=6 rats/group). AST: aspartate aminotransferase; ALT: alanine aminotransferase.

## Discussion

The present study showed, for the first time, that vitamin D_3_-induced antinociception occurs in parallel to changes in pro-oxidant and antioxidant markers in spinal cord of CCI rats, a model of neuropathic pain.

Vitamin D_3_ is a steroid hormone that is important in the regulation of plasma calcium concentration (28). Thus, it could be expected that supplementation with this vitamin would change body calcium homeostasis. However, no hypercalcemia was observed in rats after 4 weeks of being fed test diets with different doses of vitamin D (from deficiency to levels equivalent to human doses of 0.015, 0.25, and 3.75 mg/day) (29). Also, no change was found in plasma calcium concentration for rats orally fed vitamin D_3_ weekly at a dose of 500 IU/kg for 12 weeks (19). Thus, it is probable that the rats used in the present study did not show changes in body calcium homeostasis, however, further studies are necessary to clarify this suggestion.

Our neuropathic pain model reproduced the classical symptoms of decreased mechanical withdrawal threshold and thermal withdrawal latency (17,18). The administration of vitamin D_3_ attenuated changes, suggesting that this treatment induced antinociceptive effects in rats with CCI. A previous study also found antinociception in CCI rats that received vitamin D_3_ (11). Thus, our study reproduced the antinociceptive effect of the vitamin D_3_.

Vitamin D_3_ also attenuated the decrease in SFI in CCI rats. A similar result was found for the peroneal functional index of rats with the peroneal nerve cut out and autografted in an inverted position and which were orally fed vitamin D_3_ for 12 weeks (19). Since peroneal functional index values at first month were similar to those found in the present study, the vitamin D_3_ administration induced functional recovery in the two models of peripheral nerve injury.

Interestingly, attenuation of SFI occurred in parallel with improvement in mechanical withdrawal threshold and thermal withdrawal latency of CCI rats. This raises the possibility of some relationship between these results. However, CCI-induced foot deformities appear more related to spontaneous ongoing pain rather than to mechanical allodynia (30). Unfortunately, the present study did not assess spontaneous ongoing pain in CCI rats. Moreover, a previous study showed that ongoing pain-like behaviors occurred in parallel with maximal mechanical hypersensitivity in a rat model of paclitaxel-induced peripheral neuropathy (31). Thus, it is possible that vitamin D also improves ongoing pain-like behaviors in CCI rats, and that this effect may be related to the attenuation of SFI in these rats. Further research is needed to address this question.

Still, other factors need to be considered. A recent study showed that intraperitoneal administration of 25-hydroxyvitamin D_3_ for 8 weeks significantly alleviated inflammation by decreasing proinflammatory while increasing anti-inflammatory molecules (32). Vitamin D was also able to modify the expression of genes involved in axogenesis and myelination processes after peroneal nerve injury (19). Thus, these actions of vitamin D may also be contributing to attenuation of nociception and SFI.

Since vitamin D appears to have a major role in regulating ROS levels (13–15), we hypothesized that these actions of vitamin D would contribute to its antinociceptive effect and attenuation of SFI. This suggestion was based on previous research that revealed improvements in mechanical withdrawal threshold, thermal withdrawal latency, and SFI in parallel with changes in pro-oxidant and antioxidant markers in injured sciatic nerve and spinal cord of CCI rats treated with antioxidant compounds (7,17,18). In fact, and demonstrated here for first time, vitamin D_3_ administration prevented increases in SAG, H_2_O_2_, and lipid hydroperoxide levels in spinal cord of the same CCI rats that exhibited attenuation in nociception and SFI. In these rats, vitamin D_3_ also prevented marked decreases in spinal cord total thiols content and increases in lipid hydroperoxide levels in injured sciatic nerve. Thus, a relationship between antinociception and changes in spinal cord oxidative parameters cannot be discarded.

Previous research has shown that vitamin D down-regulated the nicotinamide adenine dinucleotide phosphate oxidases (NOX) that generate ROS while upregulating superoxide dismutase (14) and the expression of glutathione peroxidase (15). Superoxide dismutase converts superoxide to H_2_O_2_ and glutathione peroxidase is one of the enzymes that catalyzes the breakdown of H_2_O_2_ to H_2_O and O_2_ (3). Vitamin D was also found to regulate the expression of γ-glutamyltranspeptidase, glutamate cysteine ligase, and glutathione reductase, which contribute to the synthesis of glutathione (13). According to this author, vitamin D also increases the activity of glucose-6-phosphate dehydrogenase, a mechanism that also contributes to increased formation of glutathione, the most abundant thiol in mammals (33). Thus, it may be suggested that the decreases in SAG and H_2_O_2_ levels in spinal cord of vitamin D_3_-treated CCI rats are related to the actions of vitamin D on NOX, superoxide dismutase, and glutathione peroxidase enzymes, while the preventive effect on total thiols is related to actions of this vitamin that increase glutathione. Nevertheless, future studies need to examine the activity of these enzymes in spinal cord of vitamin D-treated CCI rats to evaluate this suggestion.

The antioxidant actions of vitamin D may be related to the lack of increase in lipid hydroperoxides in sciatic nerve and spinal cord of vitamin-treated CCI rats. However, TAC increased in spinal cord of vitamin D_3_-treated CCI rats by 28 days, but only compared to vehicle-treated CCI rats at the same time of measurement. The lack of significant changes in TAC, when comparing naive rats and vitamin-treated CCI rats, may indicate that the treatment did not disrupt the well-integrated antioxidant defense networks. However, we analyzed TAC using only trolox equivalent antioxidant capacity assay that measures albumin, uric acid, ascorbic acid, α-tocopherol, and bilirubin (34). It is recommended that various assays should be used for TAC evaluation (34). Thus, further research using a combination of TAC assays and analysis of other specific antioxidants is needed to better understand the relationship between TAC and vitamin D in nervous tissue of CCI rats.

Also, we did not assess plasma 25(OH)vitamin D levels, the major circulating form of vitamin D and the form generally used to monitor vitamin D status (35). Factors such as type and amount of dietary fiber, vitamin D status, and genetic variation in proteins involved in intestinal absorption, are suspected of affecting vitamin D bioavailability (36). A previous study suggested that arterial stiffness and systolic blood pressure both showed a U-shaped dose-response curve for vitamin D, with the lowest values (best cardiovascular health) being observed when plasma 25(OH)vitamin D levels were 43 nM in normal male Wistar rats (29). According to these authors, the level of 25(OH)vitamin D was 108 nmol/L when rats consumed a daily vitamin D dose equivalent to 5.45 µg/day. In this condition, there was a 10-mmHg increase (not significant) in systolic blood pressure, which was not found for diastolic blood pressure (29). Since our study used vitamin D_3_ at the dose of 500 IU/kg (equivalent to 12.5 µg/day), it may be suggested that the 25(OH)vitamin D levels were higher than 108 nmol/day. Thus, we cannot exclude that some changes did occur in the cardiovascular system. Still, the efficiency of an intermediate dose of vitamin D_3_ was not tested in our animal model. However, no evidence of toxicity was observed in our study, although only plasmatic ALT, AST, direct bilirubin, and creatinine levels were assessed. Also, vitamin D_3_-treated rats did not show any signs of behavioral change throughout the entire observation period, and no death occurred and there was no significant change in body weight. The lack of changes in these parameters is consistent with a previous study using rats treated weekly with vitamin D_3_ at a dose of 500 IU/kg (19). Despite limitations, our results are important because they are the first to demonstrate the effects of vitamin D on nervous tissue pro-oxidant and antioxidant biomarkers in a model of neuropathic pain. Since changes in pro-oxidant and antioxidant biomarkers in sciatic nerve and spinal cord of vehicle-treated CCI rats was in accordance with previous studies (7,8,17,18), this reinforces our results.

The reductions in mechanical threshold in sham rats may be related to the procedures involving the manipulation of deep tissues, such as muscles and adjacent connective tissue, which induce pain (17). Interestingly, there was no effect of vitamin D_3_ in these rats. Convergent and divergent mechanisms underlie inflammatory *vs* neuropathic pain that do not always appear with a clear mechanistic delineation, especially when applied to the progression from acute to chronic pain (37). This may be related to the different effects of vitamin D in sham and CCI rats.

In conclusion, the present study reproduced the antinociceptive effect of vitamin D_3_ in rats with CCI_._ It also showed, for the first time, that this treatment ameliorated SFI and modulated pro-oxidant and antioxidant markers in spinal cord of CCI rats. Since antinociception occurred in parallel to changes in pro-oxidant and antioxidant markers in spinal cord, these changes may be contributing to vitamin D-induced antinociception in CCI rats and should be considered in further studies on this question.

## References

[1] Colloca L, Ludman T, Bouhassira D, Baron R, Dickenson AH, Yarnitsky D (2017). Neuropathic pain. Nat Rev Dis Primers.

[2] Grace PM, Gaudet AD, Staikopoulos V, Maier SF, Hutchinson MR, Salvemini D (2016). Nitroxidative signaling mechanisms in pathological pain. Trends Neurosci.

[3] Poljsak B, Suput D, Milisav I (2013). Achieving the balance between ROS and antioxidants: when use the synthetic antioxidants. Oxid Med Cell Longev.

[4] Bittar A, Jun J, La JH, Wang J, Leem JW, Chung JM (2017). Reactive oxygen species affect spinal cell type-specific synaptic plasticity in a model of neuropathic pain. Pain.

[5] Sözbir E, Naziroglu M (2016). Diabetes enhances oxidative stress-induced TRPM2 channel activity and its control by N-acetylcysteine in rat dorsal root ganglion and brain. Metab Brain Dis.

[6] Przemyslaw W, Piotr K, Grazyna C, Danuta KP, Malgorzata I, Bernadeta M (2011). Total, free, and protein-bound thiols in plasma of peritoneal dialysis and predialysis patients. Int Urol Nephrol.

[7] Riffel APK, Santos MCQ, de Souza JA, Scheid T, Horst A, Kolberg C (2018). Treatment with ascorbic acid and α-tocopherol modulates oxidative-stress markers in the spinal cord of rats with neuropathic pain. Braz J Med Biol Res.

[8] Naik AK, Tandan SK, Dudhgaonka SP, Jadhav SH, Kataria M, Prakash VR (2006). Role of oxidative stress in pathophysiology of peripheral neuropathy and modulation by N-acetyl-l-cysteine in rats. Eur J Pain.

[9] de Oliveira DL, Hirotsu C, Tufik S, Andresen ML (2017). The interfaces between vitamin D, sleep and pain. J Endocrinol.

[10] Poisbeau P, Aouad M, Gazzo G, Lacaud A, Kemmel V, Landel V (2019). Cholecalciferol (vitamin D_3_) reduces rat neuropathic pain by modulating opioid signaling. Mol Neurobiol.

[11] Banafshe HR, Khoshnoud MJ, Abed A, Saghazadeh M, Mesdaghinia A (2019). Vitamin D supplementation attenuates the behavioral scores of neuropathic pain in rats. Nutr Neurosci.

[12] Kumar A, Kaur H, Singh A (2018). neuropathic pain models caused by damage to central or peripheral nervous system. Pharmacol Rep.

[13] Berridge MJ (2017). Vitamin D deficiency accelerates ageing and age-related diseases: a novel hypothesis. J Physiol.

[B14] Berridge MJ (2016). Vitamin D, reactive oxygen species and calcium signaling in ageing and disease. Philos Trans R Soc Lond B Biol Sci.

[15] Berridge MJ (2015). Vitamin D cell signaling in health and disease. Biochem Biophys Res Commun.

[16] Sepidarkish M, Farsi F, Akbari-Fakhrabadi M, Namazi N, Almasi-Hashiani A, Maleki Hagiagha A (2019). The effect of vitamin D supplementation on oxidative stress parameters: a systematic review and meta-analysis of clinical trials. Pharmacol Res.

[17] Scheid T, Moraes MS, Henriques TP, Riffel APK, Bello-Klein A, Poser GLV (2018). Effects of methanol fraction from leaves of *Schinus terebinthifolius* Raddi on nociception and spinal-cord oxidative biomarkers in rats with neuropathic pain. Evid Based Complement Alternat Med.

[18] Horst A, de Souza JA, Santos MC, Riffel AP, Kolberg C, Ribeiro MF (2017). N-acetylcysteine downregulates phosphorylated p-38 expression but does not reverse the increased superoxide anion levels in the spinal cord of rats with neuropathic pain. Braz J Med Biol Res.

[19] Chabas JF, Stephan D, Marqueste T, Garcia S, Lavaut MN, Nguyen C (2013). Cholecalciferol (vitamin D_3_) improves myelination and recovery after nerve injury. Plos One.

[20] Bennett GJ, Xie YK (1988). A peripheral mononeuropathy in rat that produces disorders of pain sensation like those seen in man. Pain.

[21] de Medinaceli L, Freed WJ, Wyatt RJ (1982). An index of the functional condition of rat sciatic nerve based on measurements made from walking tracks. Exp Neurol.

[22] Zhou Q, Bao Y, Zhang X, Zeng L, Wang L, Wang J (2014). Optimal intervals for hot water immersion tail-flick test in rats. Acta Neuropsychiatr.

[23] Erel O (2004). A novel automated direct measurement method for total antioxidant capacity using a new generation, more stable ABTS radical cation. Clin Biochem.

[24] Aksenov MY, Markesbery WR (2001). Changes in thiol content and expression of glutathione redox system genes in the hippocampus and cerebellum in Alzheimer's disease. Neurosci Lett.

[25] Jiang ZY, Woollard ACS, Wolff SP (1991). Lipid hydroperoxide measurement by oxidation of Fe^+2^ in the presence of xylenol orange. Comparison with the TBA assay and an iodometric method. Lipids.

[26] Wang HD, Pagano PJ, Du Y, Cayatte AJ, Quinn MT, Brecher P (1998). Superoxide anion from the adventitia of the rat thoracic aorta inactivates nitric oxide. Circ Res.

[27] Pick E, Keisari Y (1980). A simple colorimetric method for the measurement of hydrogen peroxide produced by cells in culture. J Immunol Methods.

[28] Hur J, Lee P, Kim MJ, Cho YW (2014). Regulatory effect of 25-hydroxyvitamin D3 on nitric oxide production in activated microglia. Korean J Physiol Pharmacol.

[29] Mirhosseini NZ, Knaus SJ, Bohaychuk K, Singh J, Vatanparast HA, Weber LP (2016). Both high and low plasma levels of 25-hydroxy vitamin D increase blood pressure in a normal rat model. Br J Nutr.

[30] Nakazato-Imasato E, Kurebayashi Y (2009). Pharmacological characteristics of the hind paw weight bearing difference induced by chronic constriction injury of the sciatic nerve in rats. Life Sci.

[31] Griffiths LA, Duggett NA, Pitcher AL, Flatters SJL (2018). Evoked and ongoing pain-like behaviours in a rat model of paclitaxel-induced peripheral neuropathy. Pain Res Manag.

[32] Han J, Cheng C, Zhu Z, Lin M, Zhang DX, Wang ZM (2019). Vitamin D reduces the serum levels of inflammatory cytokines in rat models of periodontitis and chronic obstructive pulmonary disease. J Oral Sci.

[33] Poole LB (2015). The basics of thiols and cysteines in redox biology and chemistry. Free Radic Biol Med.

[34] Rubio CP, Hernández-Ruiz J, Martinez-Subiela S, Tvarijonaviciute A, Ceron JJ (2016). Spectrophotometric assays for total antioxidant capacity (TAC) in dog serum: an update. BMC Vet Res.

[35] Tuckey RC, Cheng CYS, Slominski AT (2019). The serum vitamin D metabolome: what we know and what is still to discover. J Steroid Biochem Mol Biol.

[36] Borel P, Caillaud D, Cano NJ (2015). Vitamin D bioavailability: state of the art. Crit Rev Food Sci Nutr.

[37] Burma NE, Leduc-Pessah H, Fran CY, Trang T (2017). Animal models of chronic pain: Advances and challenges for clinical translation. J Neurosci Res.

